# Regulation of neutrophil associated RNASET2 expression in rheumatoid arthritis

**DOI:** 10.1038/s41598-024-77694-y

**Published:** 2024-11-05

**Authors:** Mauro Passari, Sara Scutera, Tiziana Schioppa, Laura Tiberio, Silvia Piantoni, Nicola Tamassia, Mattia Bugatti, William Vermi, Fabrizio Angeli, Alessia Caproli, Valentina Salvi, Francesca Sozio, Angela Gismondi, Helena Stabile, Franco Franceschini, Daniela Bosisio, Francesco Acquati, Sonja Vermeren, Silvano Sozzani, Laura Andreoli, Annalisa Del Prete, Tiziana Musso

**Affiliations:** 1 Department of Molecular and Translational Medicine, University of Brescia, Viale Europa 11, Brescia, 25123 Italy; 2 Department of Public Health and Pediatrics, University of Turin, Turin, Italy; 3 IRCCS Humanitas Research Hospital-Rozzano, Milan, Italy; 4 Department of Clinical and Experimental Sciences, Unit of Rheumatology and Clinical Immunology - ASST, University of Brescia, Spedali Civili of Brescia, Brescia, Italy; 5 Department of Medicine, Section of General Pathology, University of Verona, Verona, Italy; 6 Department of Molecular Medicine, Laboratory Affiliated to Istituto Pasteur Italia- Fondazione Cenci Bolognetti, Sapienza University of Rome, Rome, Italy; 7 Human Genetics Laboratory, Department of Biotechnology and Life Sciences, University of Insubria, Varese, Italy; 8 Centre for Inflammation Research, Institute for Regeneration and Repair, The University of Edinburgh, Edinburgh, UK

**Keywords:** Immunology, Inflammation, Innate immune cells, Innate immunity

## Abstract

Neutrophils (PMNs) are key players of innate immune responses through the release of cytoplasmic granule content and the formation of neutrophil extracellular traps (NETs). RNASET2 is an acidic ribonuclease, recently proposed as an alarmin signal associated with inflammatory responses. Here we show that, along the neutrophil maturation cascade, RNASET2 is expressed in segmented and mature PMNs. In human PMNs, RNASET2 colocalized with primary and tertiary granules and was found to be associated with NETs following PMA or Nigericin stimulation. Similarly, activation of PMNs by soluble immune complexes, a hallmark of several autoimmune diseases, also induced RNASET2-associated NETs. Genome-wide association studies recently identified RNASET2 among a cluster of genes associated with increased susceptibility to develop autoimmune diseases, including rheumatoid arthritis (RA). RNASET2 was found expressed by PMNs and macrophages infiltrating inflamed joints in a murine model of RA (K/BxN Serum-Transfer-Induced Arthritis, STIA), by immunostaining. Similar results were found in synovial biopsies of RA patients with active disease. In addition, we demonstrate that RNASET2 circulating levels correlated with the onset and the severity of disease in two mouse models of inflammatory arthritis, STIA and CIA (Collagen-Induced Arthritis) and in serum of RA patients. These results show that PMNs are an important source of RNASET2 and that its circulating levels are associated with RA development suggesting a role for RNASET2 in the pathogenesis of immune-mediated diseases.

**Supplementary Information:**

The online version contains supplementary material available at 10.1038/s41598-024-77694-y.

## Introduction

Neutrophils (PMNs) are key players of innate immune responses, representing the first line of host defence against invading pathogens and tissue injury^1^. PMNs, the most abundant circulating leukocytes, are rapidly recruited at the sites of infection or damage, where they exert essential antimicrobial activities and promote the resolution of inflammation and tissue repair^2^. Neutrophil effector mechanisms include the release of proinflammatory cytokines, reactive oxygen and nitrogen species (ROS and RNS), and granules containing degradative enzymes, which can cause further damage to the tissue and amplify the neutrophilic response^3^. Moreover, in response to certain stimuli PMNs can extrude neutrophil extracellular traps (NETs), web-like structures made of DNA and associated cytoplasmic and granular proteins^4^. There is growing evidence that the process of NET formation, also referred to as NETosis and originally described as a mechanism fighting bacterial infection, is associated with the pathogenesis and development of autoimmune diseases such as rheumatoid arthritis (RA)^5^.

RNASET2 encodes for the only mammalian member of the T2 family of RNases and includes both intracellular and secretory forms^6^. The main biological role of intracellular RNASET2 is to cleave or degrade exogenous or endogenous RNA substrates and to contribute to mitochondrial RNA metabolism^6^. The secretory RNASET2 form plays immunomodulatory and antimicrobial roles^7^. Since tissue damage and oxidative stress can induce RNASET2 secretion, several reports candidate it as stress response gene^8,9^. Indeed, RNASET2 secretion following tissue injury could act as “alarmin signal” able to activate innate immunity and regulate the host responses and tissue remodelling^10^. RNASET2 is involved in multiple physiological processes, such as angiogenesis, apoptosis, cell proliferation/differentiation, and immunomodulation^6^. Moreover, dysregulation of RNASET2 expression has been associated with several pathological conditions including autoimmune diseases and cancer^11,12^.

In a recent transcriptomic analysis of immune cell gene expression profiles, neutrophils were reported to express the highest levels of RNASET2^13^. However, its regulation, release, and expression pattern in neutrophil-related physiopathological conditions remains yet to be deciphered. Moreover, recent genome-wide association studies (GWAS) identified RNASET2 among novel genes associated with increased susceptibility to develop autoimmune diseases, including RA^14,15^. In RA, PMNs are the most abundant leukocytes infiltrating inflamed joints, and the importance of these cells in the initiation and progression of the disease has been demonstrated in humans as well as in murine models^16,17^. Neutrophil activation in RA is associated with enhanced NET formation driven by soluble factors and immunocomplexes (IC)^18,19^.

Here we report the expression pattern of RNASET2 during neutrophil maturation, the localization of RNASET2 in primary and tertiary granules and the association of RNASET2 with NET formation. In addition, the possible involvement of RNASET2 in the pathogenesis of RA was investigated in two murine models of inflammatory arthritis and in patients with active disease. Taken together, these results propose a role for RNASET2 in the pathogenesis of autoimmune diseases.

## Results

### RNASET2 expression and intracellular localization in human neutrophils

**Fig. 1:**
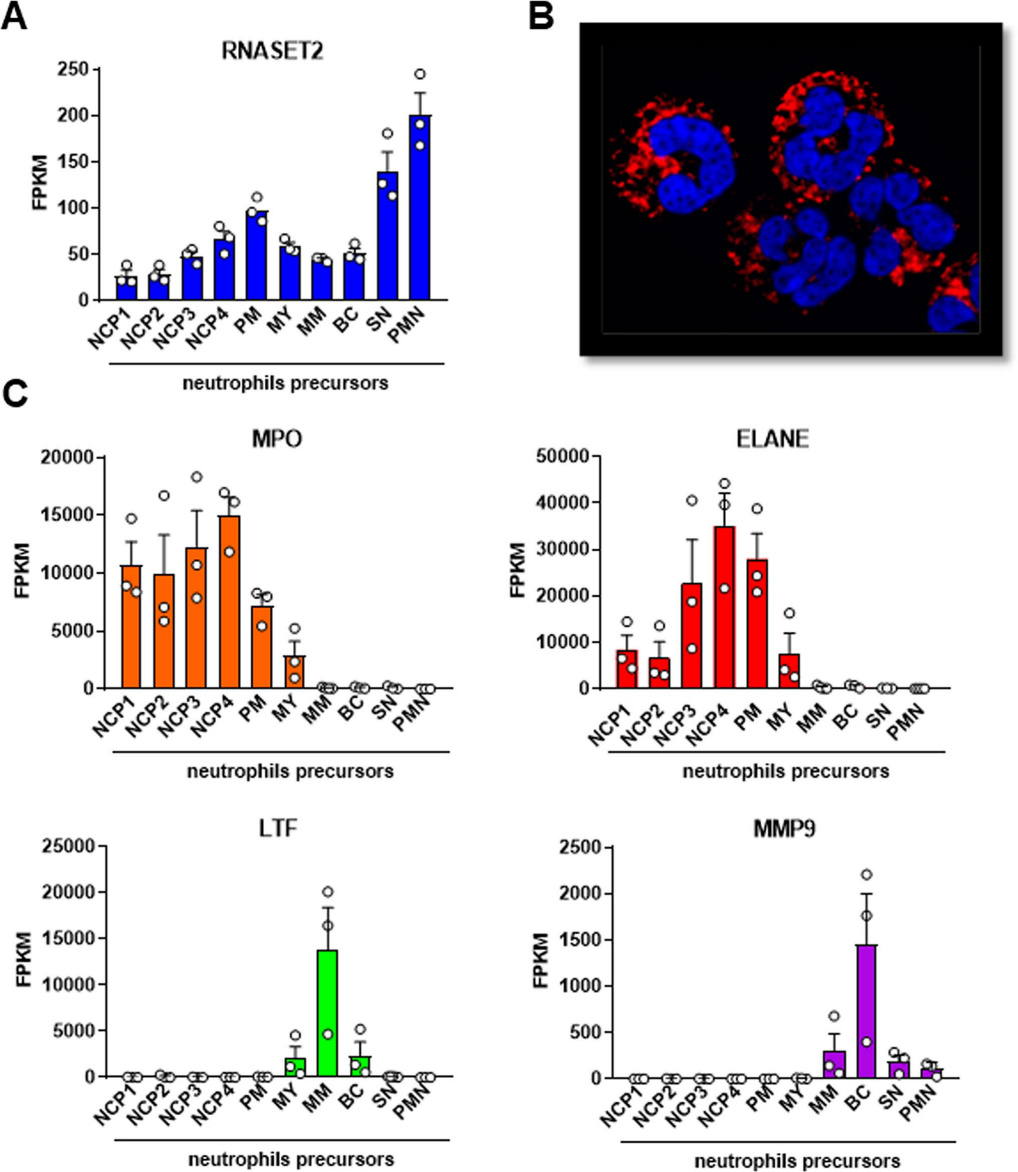
Transcriptomic analysis and intracellular localization of RNASET2 in human neutrophils – RNAseq analysis of (**A**) *RNASET2* and (**C**) selected neutrophil granule proteins (*MPO*, *ELANE*, *LTF*, and *MMP9*) transcript levels in Neutrophil Committed Precursors (NCP)1, NCP2, NCP3, NCP4, promyelocyte (PM), myelocytes (MY), metamyelocyte (MM), Band cells (BC), Segmented Neutrophils (SN) and circulating blood-derived neutrophil (PMN). Values represent the mean ± SEM of FPKM (Fragments Per Kilobase per Million mapped fragments). (**B**) Representative immunofluorescence image of human neutrophils showing RNASET2 reactivity (red) and nuclei staining with DAPI (blue). Magnification, 1000x.

Neutrophils are involved in the initiation and perpetuation of inflammation^1^ and were shown to express the higher number of RNASET2 transcripts in comparison to the other immune cells^13^. To investigate the RNASET2 expression during neutrophil maturation, we analysed the transcriptomes of circulating human neutrophils and their bone marrow progenitors, including Neutrophil Committed Precursors (NCPs), promyelocyte (PM), myelocytes (MY), metamyelocyte (MM), band cells (BCs) and segmented neutrophils (SN), retrieved from a recent study^20^. We found that the RNASET2 expression pattern along the neutrophil maturation cascade (Fig. 1A) was remarkably different from those of genes encoding for typical primary (*MPO* and *ELANE*), secondary (*LTF*) and tertiary (*MMP9*) granule proteins (Fig. 1C). As expected^20^, *MPO* and *ELANE* showed the highest expression levels at the earliest stages of neutrophil maturation (NCPs and PM) while *LTF* and *MMP9* expression was higher at later stages (MM and BC, respectively) (Fig. 1C). Unlike typical genes encoding for granule proteins, RNASET2 was expressed at all neutrophil maturation phases, peaking in PM, therefore similarly to primary granule proteins, and in SN and mature PMNs (Fig. 1A). Immunofluorescence of human neutrophils revealed that RNASET2 reactivity presented a granular staining pattern, suggesting that, at the protein level, RNASET2 was stored within neutrophil granules (Fig. 1B).

**Fig. 2:**
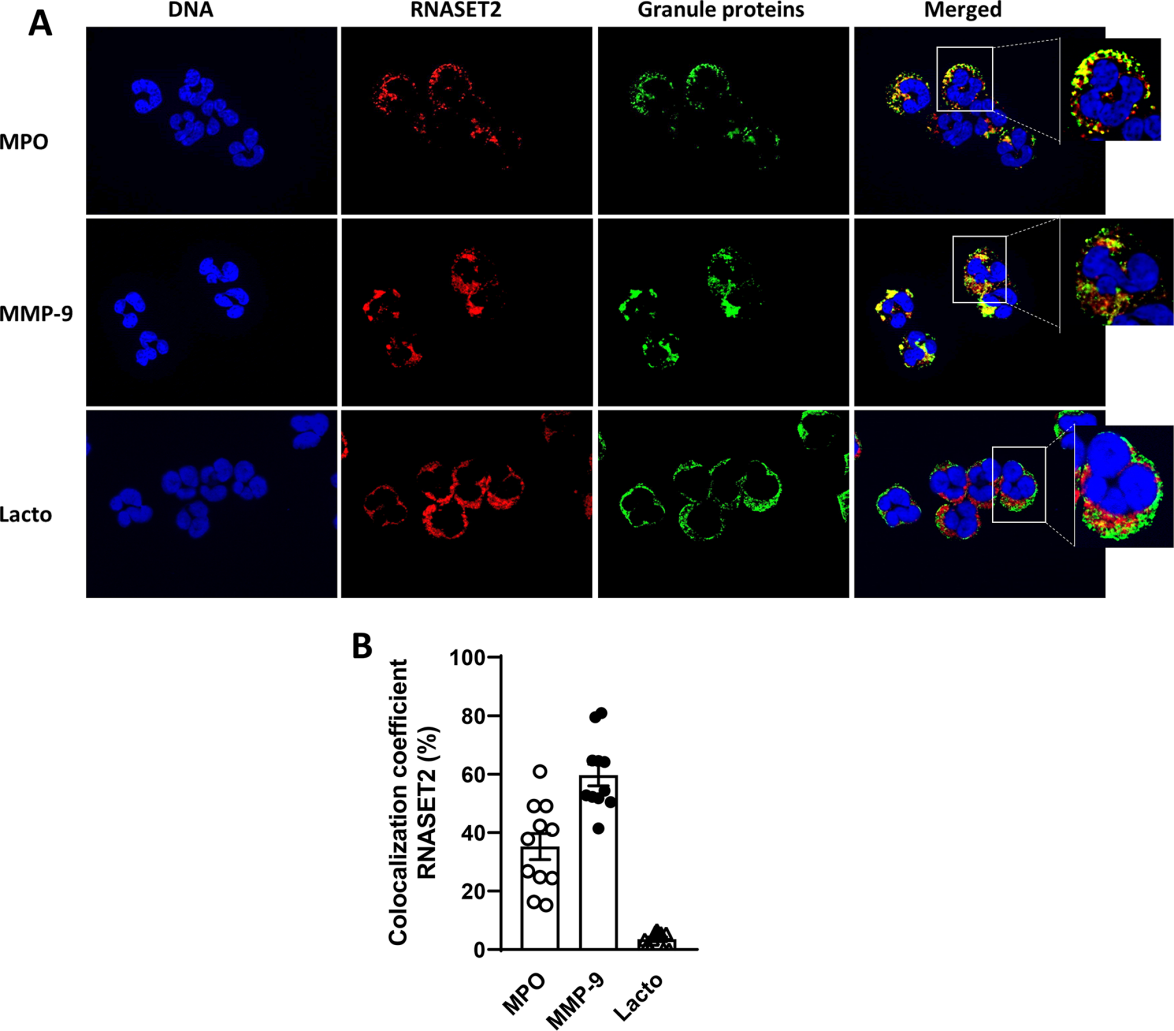
Colocalization of RNASET2 with granule proteins in human neutrophils – (**A**) Immunofluorescent co-localization of RNASET2 with neutrophil granule proteins. Double immunofluorescent staining for RNASET2 (red) and myeloperoxidase (MPO), or Lactoferrin or metalloproteinase-9 (MMP-9) (green). Magnification, 1000x. (**B**) Percentage of colocalization of RNASET2 with myeloperoxidase (MPO), metallo-proteinase-9 (MMP-9) and lactoferrin (lacto). Each dot represents the colocalization percentage assessed in one high magnification (1000X) field. Colocalization data are expressed as mean ± SEM of the colocalization percentage in one representative sample.

The cytoplasmic granules of neutrophils contain various serine proteases which can be released following stimulation and can contribute to autoimmune disease-associated tissue damage, such as in RA^21^. Primary granules are the main storage of the most pro-catabolic mediators, including elastase, myeloperoxidase, cathepsins and defensins, whereas the secondary and tertiary granules contain lactoferrin and matrix metalloprotease 9 (MMP9), respectively. To better characterize the intracellular localization of RNASET2 we performed a double immunofluorescence (IF) analysis of human neutrophils. We used three different markers to identify granules in the cytoplasm: MPO (for primary granules), lactoferrin (for secondary granules) and MMP9, (for tertiary granules). Double immunofluorescent signal showed that RNASET2 reactivity mainly colocalized with MPO and MMP9 signals (Fig. 2A, upper and middle panels). No co-localization with lactoferrin was observed (Fig. 2A, lower panels). These results were confirmed by the quantification of colocalization coefficient as shown in Fig. 2B (RNASET2/MPO; 35.23 ± 4.46; RNASET2/MMP-9: 59.69 ± 3.72; RNASET2/Lactoferrin: 3.51 ± 0.76). Overall, these findings suggest that RNASET2 in human neutrophils colocalizes with primary and tertiary granules, but no colocalization was found with secondary ones.

## RNASET2 is associated with neutrophil extracellular traps (NETs)

**Fig. 3:**
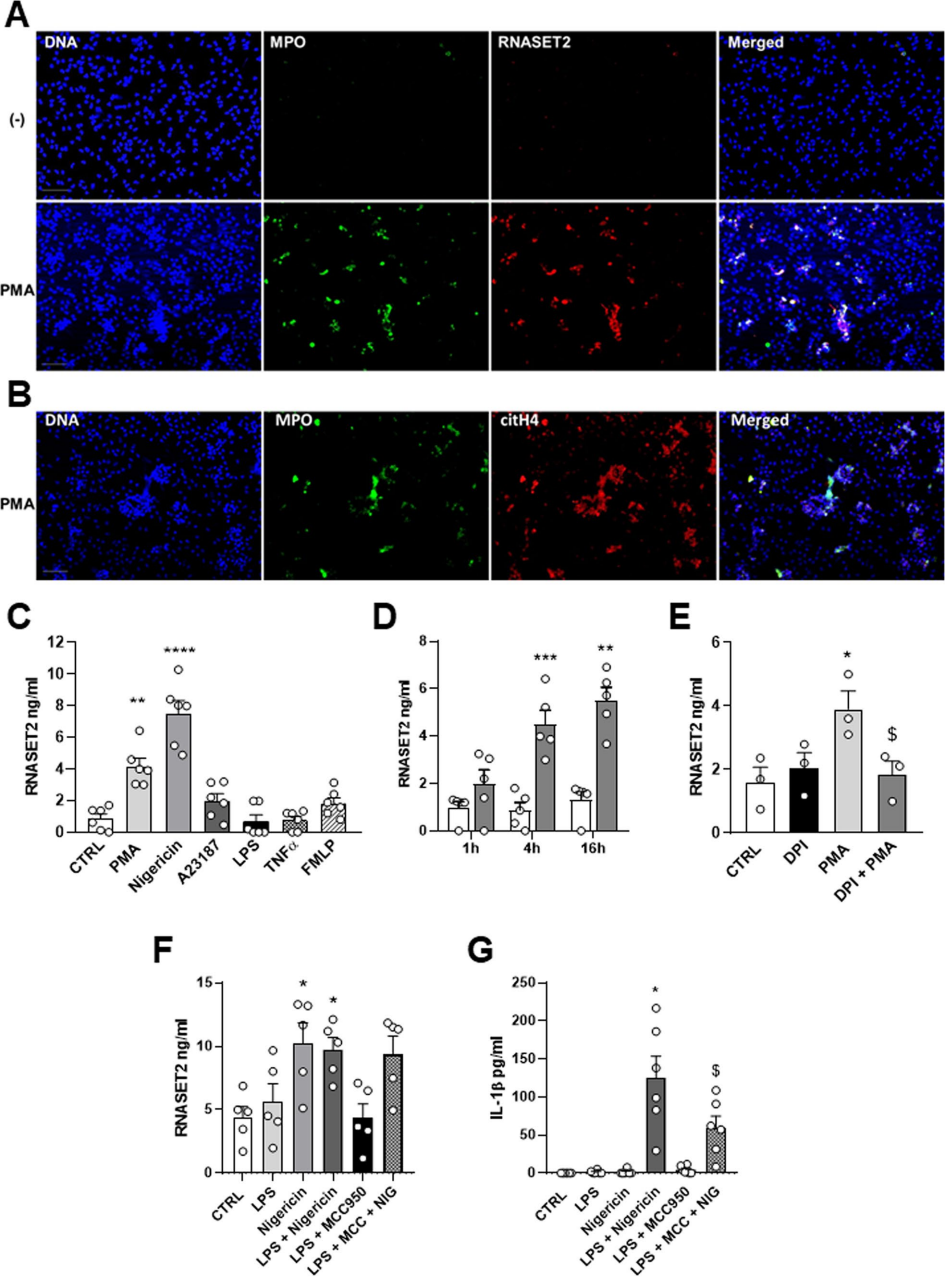
RNASET2 release by NET-inducer stimuli in human neutrophils – (**A**) Identification of NET-associated RNASET2 expression (red) by co-localization (merged) with typical NET structures, namely MPO (green) and DNA (blue). (**B**) Co-localization of DNA (blue), MPO (green) with citrullinated histone 4 (citH4, red). (**C**) In vitro RNASET2 release by ELISA assay in response to several stimuli, including PMA, Nigericin, A23187, TNFα, and fMLP, **p > 0.01, PMA vs. CTRL; ****P < 0.0001 Nigericin vs. CTRL, by one-way ANOVA. (**D**) Kinetics of RNASET2 release following 1 h, 4 h and 16 h PMA stimulation. ***p < 0.001, PMA 4 h and **p < 0.01 PMA 16 h vs. related CTRL, by one-way ANOVA. (**E**) Inhibition of RNASET2 levels by NADPH inhibitor DPI, *p < 0.05, PMA vs. CTRL, § *p* < 0.05, PMA vs. PMA + DPI, by one-way ANOVA. (**F**) RNASET2 release following LPS priming. *p < 0.05, Nigericin and LPS + Nigericin vs. CTRL, by one-way ANOVA. (**G**) IL-1β release needs LPS-priming and is inhibited by the inflammasome inhibitor MCC950. *p < 0.05 LPS + Nigericin vs. CTRL, and §*p* < 0.05 LPS + Nigericin vs. LPS + Nigericin + MCC by one-way ANOVA.

Neutrophil extracellular traps (NETs) have been recently recognized as a potent weapon of the defence strategy of neutrophils. Considering the potential role of NETs in driving the pathogenesis of autoimmune and inflammatory disorders^22^, we asked whether RNASET2 could be associated with the release of DNA extracellular traps by neutrophils. To accomplish this goal, we evaluated the presence of this molecule in web-like NET structures by fluorescence microscopy. No RNASET2 positivity was detected in unstimulated cells (Fig. 3A, upper panels). On the contrary, NET formation was induced by treating for 4 h purified healthy neutrophils with PMA, the most effective recognized NET inducer. Figure 3A (lower panels) shows that PMA stimulation was able to induce the extrusion of neutrophil DNA, which colocalized with RNASET2 staining. As positive control of NET release, the citrullinated histone 4 (citH4) staining was also performed, showing a strong reactivity colocalizing with extruded DNA (Fig. 3B).

We next evaluated whether neutrophils release RNASET2 upon stimulation with various agonists engaging different activation pathways. A strong increase in RNASET2 levels compared to control was observed after 4 h treatment with PMA and Nigericin (Fig. 3C), very well-known NET inducers^4^. Treatment with the calcium ionophore A23187, the pro-inflammatory stimuli LPS and TNFα or the chemotactic factor fMLP, did not significantly influence RNASET2 release (Fig. 3C). Kinetics of PMA-induced secretion showed that, as compared with control, RNASET2 release significantly increased 4 h after stimulation and remained constant up to 16 h (Fig. 3D). Since PMA can trigger a Reactive Oxygen Species (ROS)-dependent pathway, we examined the role of redox in the secretion of RNASET2 following PMA stimulation in the presence of the NADPH oxidase inhibitor diphenyleneiodonium chloride (DPI). DPI did not reduce the secretion of RNASET2 by unstimulated cells. In contrast, DPI inhibited RNASET2 secretion by around 50%, in the presence of PMA, suggesting that redox status may play a role in the secretion of RNASET2 (Fig. 3E).

Nigericin is a potassium ionophore known also to be a potent NLRP3 activator, that similarly to other secondary stimuli, such as ATP and bacteria pore-forming toxins, can promote IL-1β release in LPS stimulating cells^23,24^. To determine whether the addition of nigericin can lead to a robust RNASET2 release in primed cells, PMN were stimulated with LPS for 2 h, followed by treatment with nigericin; RNASET2 and IL-1β concentrations were measured in the supernatants. As shown in Fig. 3F, Nigericin induced RNASET2 in both primed and unprimed cells, while it only induced IL-1β release in primed cells (Fig. 3G) ruling out a role for NLRP3 inflammasome activation in RNASET2 secretion^25^. Pretreatment with MCC950, a selective small NLRP3 inhibitor, abrogated the IL-1β response to LPS by PMNs but was ineffective to inhibit RNASET2 release, thus confirming that RNASET2 release is independent of NLRP3 inflammasome activation.

## RNASET2 is released by IC-activated human neutrophils

**Fig. 4:**
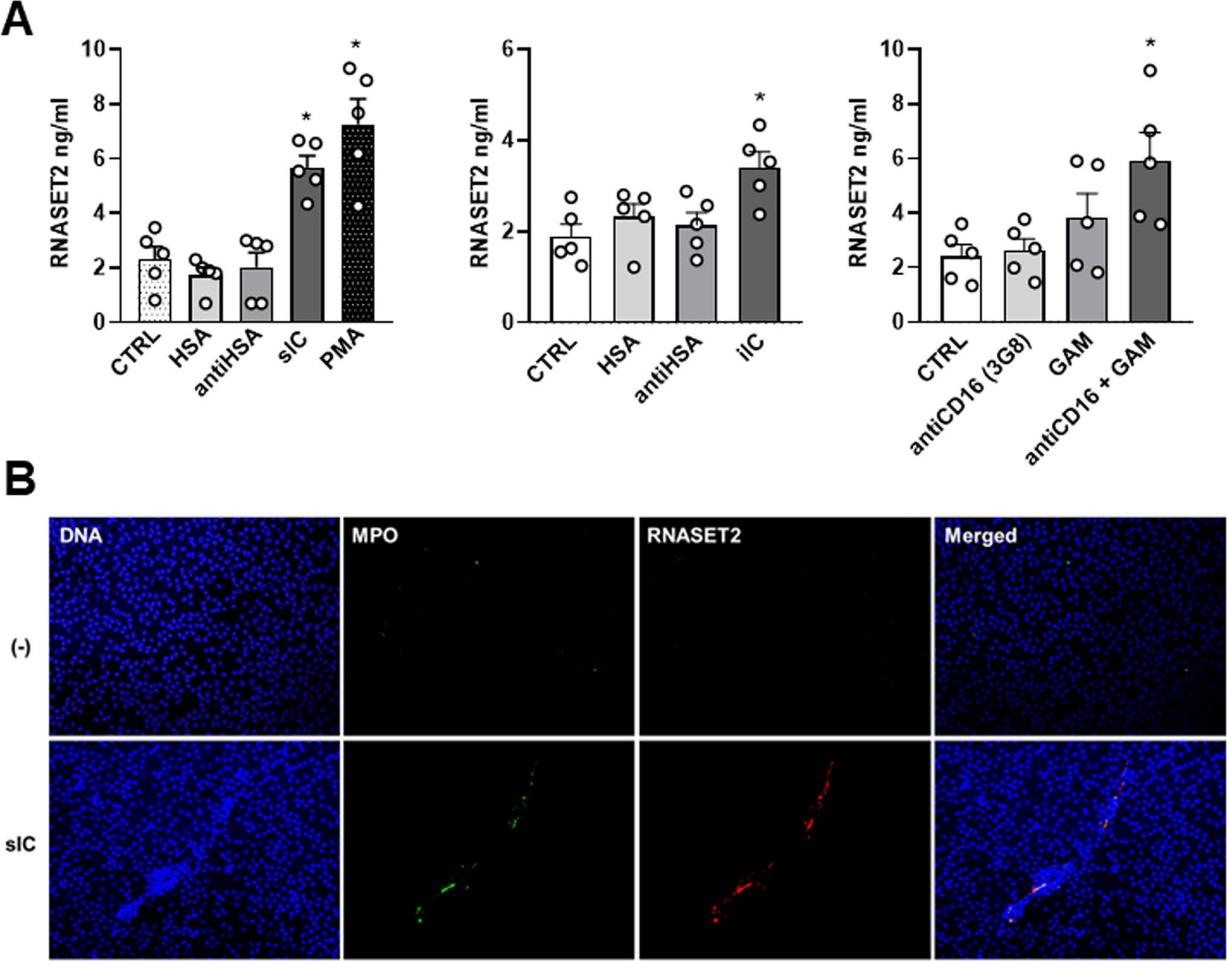
RNASET2 release by IC-activated neutrophils and CD16 cross-linking – (**A**), left panel RNASET2 release upon HSA, anti-HSA, sIC engagement, and PMA stimulated neutrophils. *p < 0.05, sIC and PMA vs. CTRL by one-way ANOVA. (Middle panel), RNASET2 release induced by iIC-activated neutrophils. *p < 0.05 iIC vs. CTRL by one-way ANOVA. (Right panel), RNASET2 release by neutrophils stimulated with anti-CD16 (clone 3G8) mAb plus F(ab′)_2_ goat anti-mouse Ig (GAM). *p < 0.05 anti-CD16 + GAM vs. CTRL by one-way ANOVA. (**B**) Colocalization (merged) of RNASET2 with DNA (blue) and MPO (green) in typical NET structure of sIC-activated PMN.

Activation of neutrophils through immune complexes (IC) plays a central role in the pathogenesis of many autoimmune inflammatory diseases and IC-mediated Fc receptor engagement is involved in local tissue damage^16,26,27^. For instance, in RA both insoluble and soluble IC can accumulate in the blood stream and in other body fluids, including synovial fluid^28^. IC can also precipitate on biological surface, such as synovial lining, and are referred as iIC, known to contribute to neutrophil inflammation^29^. Moreover, the different types of IC showed different capacity in eliciting the neutrophil effector functions, such as the induction of NET formation^30^. Since different types of IC can be reproduced in vitro depending on antigen: antibody ratio, human neutrophils were stimulated with IC prepared from human serum albumin (HSA) and rabbit polyclonal IgG to HSA (anti-HSA) to evaluate the capacity to induce RNASET2 release. Figure 4A (left panel) shows that sIC-activated neutrophils were able to induce RNASET2 release at levels that were comparable to PMA induction. No effects were observed in neutrophils stimulated by insoluble IC (1.46 *±* 0.37 vs. 2.32 *±* 0.046 ng/ml of unstimulated neutrophils), while iIC were able to elicit a significant increase of RNASET2 release over control (Fig. 4A, middle panel), although with a lower efficacy compared to sIC. Among the different IC types, the soluble ones were described to be more prone in inducing NET production. Figure 4B shows the colocalization of RNASET2 with DNA and MPO in typical web-like structure, suggesting that sIC-stimulated neutrophils induce RNASET2 release associated with NET structures.

Considering the pivotal role of FcγRIIIb (CD16) in mediating the effector functions of neutrophils in response to IC^31^, we tested if cross-linking of human CD16 was able to induce RNASET2 release. When human neutrophils were stimulated with anti-CD16 (clone 3G8) mAb plus F(ab′)2 goat anti-mouse Ig (GAM) for 4 h, we could detect a significant increase of RNASET2 secretion compared with control neutrophils (Fig. 4A, right panel). These results suggest that CD16 ligation on human neutrophils is an effective stimulus for RNASET2 release.

## Synovial and circulating expression of RNASET2 in mouse experimental RA models

Accumulating evidence suggests the involvement of neutrophils in the initiation and perpetuation of local inflammation in RA^16^, which showed dysregulated activation culminating with NET formation^32^. Considering the association of RNASET2 with NETs induced by different stimuli and the role of IC-mediated Fc receptor engagement in the pathogenesis of RA, we evaluated the local expression of RNASET2 in the STIA experimental model, a transient and acute RA model where neutrophils represent the most important effector cells^33^.

**Fig. 5:**
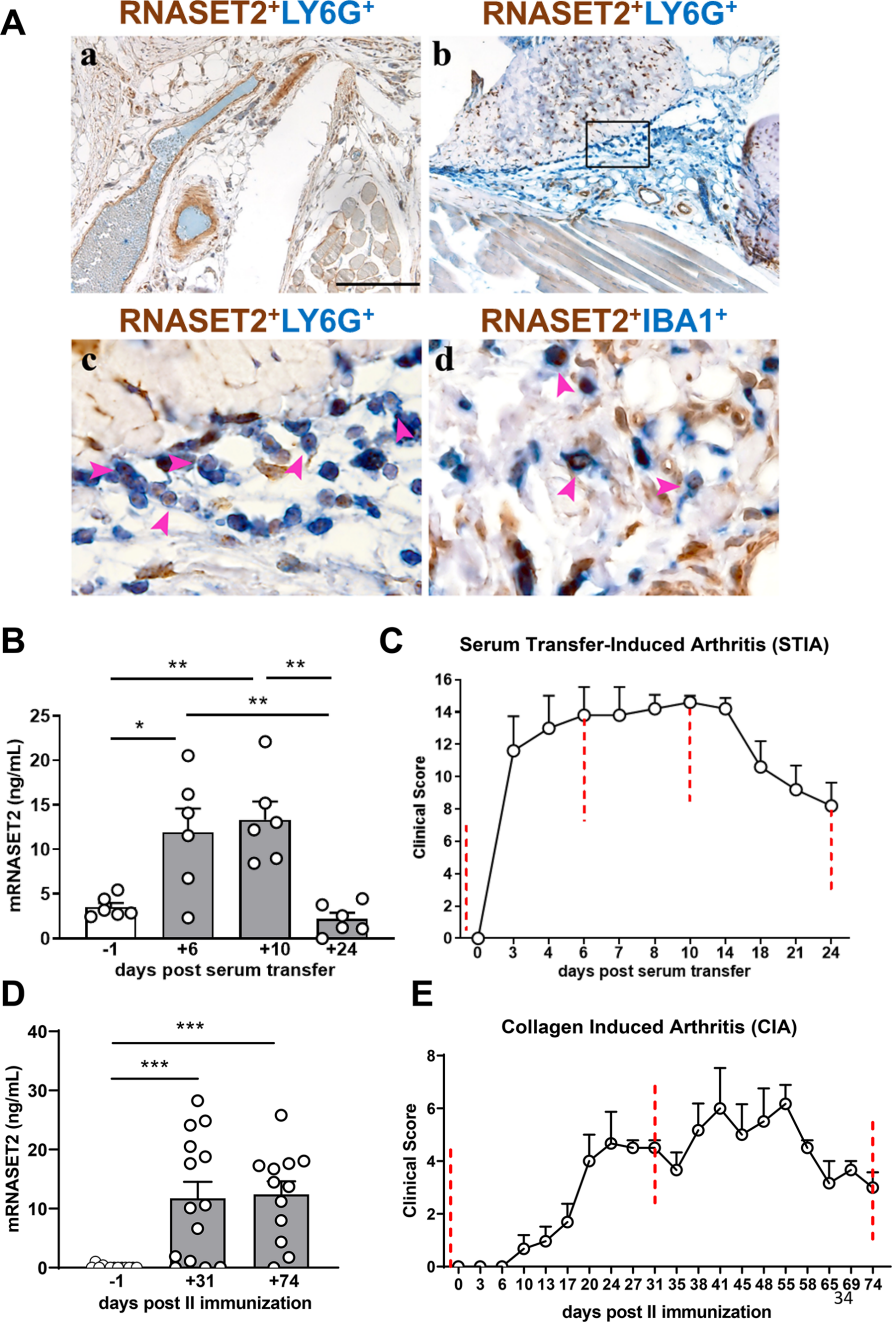
RNASET2 expression in murine models of experimental arthritis – (**A**) Representative FFPE joint samples of control (a) and STIA-induced mouse (b-d) showing lack of infiltrating cells and abundant neutrophils infiltration, respectively. Double staining of RNASET2 expression in Ly6G^+^ neutrophils (c) and IBA1^+^ macrophages (d). Magnification 100x, scale bar 200 μm (a-b); 600x, scale bar 33 μm (c-d). (**B**) Serum levels of mouse RNASET2 evaluated at different time points of STIA progression. *p < 0.05 day + 6 vs. day − 1, respectively; **p < 0.01, day + 6 and day + 10 vs. day + 24, by one-way ANOVA. *p < 0.05, day + 6 vs. day − 1, **p < 0.01 day + 10 vs. day − 1, and day + 6 vs. day + 24, and day + 10 vs. day + 24, by one-way ANOVA. Data are shown as mean ± SEM (*n* = 6 mice/group) of one representative experiment out of three. (**C**) Clinical score of STIA, induced by injection of 150 µL of K/BxN serum in C57BL/6J mice at day 0 (*n* = 6 mice/group). Scores from 4 paws were combined for each mouse, and total severity score for the group was divided by the number of arthritic mice to obtain an average severity score (clinical score, 0–16 in the 4 paws). (**D**) Serum levels of mouse RNASET2 evaluated at different time points of CIA progression. *** *p* < 0.001, day + 31 vs. day − 1, and day + 74 vs. day-1, by one-way ANOVA. (**E**) Clinical score of CIA in DBA1 mice immunized with bovine type II collagen. Scores from 4 paws were combined for each mouse, and total severity score for the group was divided by the number of arthritic mice to obtain an average severity score (clinical score, 0–16 in the 4 paws). Data are shown as mean ± SEM (*n* = 12–14 mice/group) of one representative experiment out of two.

Sections of joint tissue from arthritic animals at day + 6, corresponding to the peak of acute phase of the disease post serum-injection, were collected and assessed for studying the RNASET2 localization by immunohistochemistry analysis. While no RNASET2 positivity was observed in joint section derived from untreated mice (Fig. 5A, panel a), a strong RNASET2 expression was detected in association with the immune cells infiltrating arthritic synovium of a joint at the peak of the clinical score (Fig. 5A, panel b). By performing immunostaining, we observed that RNASET2 immunoreactivity paralleled the co-staining with Ly6G positive murine neutrophils (Fig. 5A, panel c), and IBA1 positive infiltrating murine macrophages (Fig. 5, panel d).

In addition, the circulating levels of RNASET2 in the sera of arthritic mice were evaluated at different stages of the disease. At day + 6 and + 10 after K/BxN serum injection, we found a statistically significant increase of RNASET2 serum concentration compared to serum levels observed at day − 1 (*p* < 0.05, 11.91 *±* 2.684 ng/mL vs. 3.514 *±* 0.479 ng/mL, day + 6 vs. day − 1; *p* < 0.01, 13.33 *±* 2.037 ng/mL vs. 3.514 *±* 0.479 ng/mL, day + 10 vs. day − 1) (Fig. 5B). The highest circulating levels of RNASET2 coincided with the peak of clinical signs of the disease, as assessed by clinical score (Fig. 5C). Notably, RNASET2 tended to decrease at the end of the effector phase, recapitulating the clinical features of the experimental model^34^.

To gain insights into the potential involvement of RNASET2 in the pathogenesis of RA, another experimental model of RA was used, the CIA, which is focused on both the priming phase of the disease, triggered by the activation of immune responses against type II collagen, and the effector and chronic phase, characterized by local inflammation and cartilage and joint damage^35^. Sera were collected before the induction of the disease (day − 1), at the peak of the disease (day + 31), and during the chronic phase (day + 74). In CIA model, at day + 31 and + 74 after arthritis induction, we detected significantly higher serum RNASET2 concentration as compared to baseline (*p* < 0.01, 11.75 *±* 2.783 ng/mL vs. 0.89 *±* 0.786 ng/mL, day + 31 vs. day − 1; *p* < 0.01, 12.41 *±* 2.202 ng/mL vs. 0.89 *±* 0.786 ng/mL, day + 74 vs. day − 1) (Fig. 5D). Interestingly, RNASET2 expression strongly paralleled the clinical score determined along the timeline (Fig. 5E). Indeed, circulating levels of RNASET2 remained elevated at day + 74 mirroring the clinical features of chronic phase of the disease.

The increased circulating and local expression levels of RNASET2 found in the experimental RA models strongly suggest a potential contribution of neutrophil-associated RNASET2 in the pathophysiological mechanisms accompanying the RA development.

## Systemic and local expression of RNASET2 in a cohort of RA patients

**Fig. 6:**
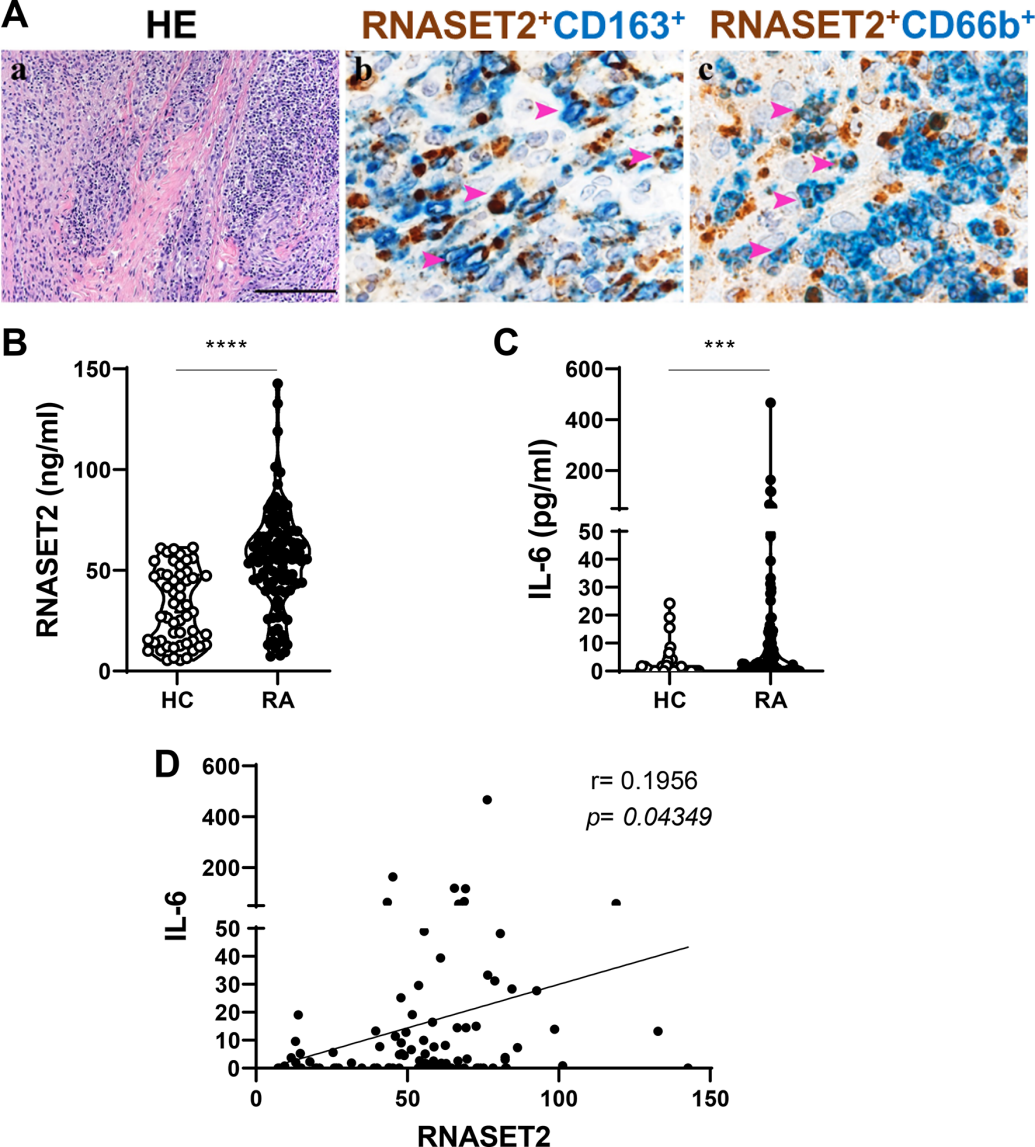
RNASET2 in RA patients. (**A**) Sections from human synovial tissue affected by RA and stained as labelled (a-c): synovitis characterized by high inflammatory infiltrate (a); RNASET2 expression found in CD163^+^ macrophages (b) and in CD66b^+^ neutrophils (c). Magnification: 100x, scale bar 200 μm (a); 600x, scale bar 33 μm (b-c). (**B**) Serum levels of RNASET2 in patients (RA) (*n* = 109) and controls (HC) (*n* = 53) detected by ELISA assay. ****p < 0.0001 by Mann Whitney U test. (**C**) Serum levels of IL-6 in patients (RA) (*n* = 109) and controls (HC) (*n* = 53) detected by ELISA assay. ***p = 0.0002 by Mann Whitney U test. (**D**) Correlation between IL-6 and RNASET2 by Spearman test (*p* = 0.04349).

To understand the potential relationship between RNASET2 synovial expression and the histopathological features of human synovial RA tissue, we performed IHC staining and RNASET2 immunoreactivity analysis of the synovial biopsy of a RA patient with active disease, characterized by a strong inflammatory infiltrate (Fig. 6A, panel a). As in inflamed mouse joints, double immunofluorescence staining showed that, in addition to RNASET2 immune reactivity colocalized with CD163-positive macrophages (panel b), CD66b^+^ neutrophils infiltrating arthritic joint expressed RNASET2 (panel c).

Based on the correlation of clinical disease features with the serum levels of RNASET2 in the murine experimental models, we examined the circulating expression of RNASET2 in a cohort of RA patients. Serum levels of RNASET2 were significantly increased in RA patients as compared with HC [56.07 (43.65–67.89) vs. 29.31 (13.96–47.68) ng/mL; *p* < 0.0001] [median (IQR)] (Fig. 6B). However, no correlation of RNASET2 serum levels with CRP nor with disease activity DAS28-CRP score was observed (Supplementary Fig. 1A and B), probably due to our patients’ features, all undergone to multiple conventional synthetic (cs)- and biologic/targeted synthetic (b/ts) Disease-Modifying Anti-Rheumatic Drugs (DMARDs). In addition, we tested our patient cohort for the circulating levels of IL-6, a pleiotropic pro-inflammatory cytokine which plays an important role in RA and its comorbidities (Fig. 6C). Serum levels of IL-6 resulted significantly increased in our RA patients as compared with HC [1.770 (0.00–12.83) vs. 0.00 (0.00–1.670) pg/mL; *p* = 0.0002] [median (IQR)]. Serum concentration of RNASET2 positively correlated with the circulating levels of IL-6 in RA patients (Fig. 6D).

Notwithstanding the limitations of our study, this is the first observation of increased circulating expression levels of RNASET2 in RA, which, together with the synovial localization of RNASET2 in the inflammatory infiltrate, strongly suggests its potential involvement in the immune-mediated regulation of joint and systemic autoimmune disease.

## Discussion

Here, we demonstrated that RNASET2 is stored in primary and tertiary granules of human PMNs and is externalized in association with NETs. We also showed that RNASET2 is expressed by the PMN infiltrating RA synovial tissue and that RNASET2 serum levels are elevated in both mouse RA experimental models and RA patients.

Our transcriptomic analysis of circulating neutrophils and their progenitor populations revealed an increased RNASET2 mRNA expression in PM and in mature neutrophils. The delayed expression of RNASET2 during neutrophil maturation is described to be shared by genes involved in immune functions, including TLR8^36^, which has been recently identified as a sensor for RNASET2 degradation products^37^. RNASET2 has been shown to cleave single-stranded RNA (ssRNA), preferentially targeting purine-uridine motifs, generating ligands that bind to different sites on TLR8 and ultimately leading to its activation^37^. Recently, GU-rich ssRNA oligonucleotides derived from the SARS-CoV-2 genome were shown to promote in human neutrophils cytokine production, apoptosis delay and a transcriptional reprogramming, characterized by the induction of thousands of proinflammatory genes^38^. Therefore, these findings, suggest that RNASET2 may perform also in human neutrophils the same recognition and activation functions observed by Greulich et al.^37^.

By confocal analysis we demonstrated that RNASET2 localizes with MPO and MMP9 in primary and tertiary neutrophil granules. Consistent with our observation, RNASET2 was identified by Rørvig et al. in neutrophil subcellular proteome profiles among proteins located mainly in primary granules and partially, to a lesser extent, in tertiary granules^39^.

Neutrophils can externalize their granule contents through degranulation or NET formation, which has been implicated in the generation of autoimmune reactions^40^. A large variety of infectious and sterile inflammatory stimuli have been described as triggers of PMN NETosis, that, based on NADPH requirement, can be divided into “suicidal” and “vital” NETosis. “Suicidal” NETosis, usually triggered in vitro by PMA, is commonly observed in inflammatory diseases such as RA, lupus and vasculitis in which autoantibodies target released NETs^41,42^. Instead, “vital” NETosis is NADPH-independent, characterized by rise in intracellular calcium concentration and is induced by calcium ionophore A23187 or specific microorganisms or lipopolysaccharides (LPS) directly or indirectly through TLR. Our findings show that RNASET2 is released and found associated to NETs, following stimulation with PMA, the best studied inducer of the NADPH-oxidase dependent NET formation in classical “suicidal” NETosis^43^. Moreover, the inhibition of NADPH oxidase with DPI, previously shown to abolish NET production^44^, effectively reduced RNASET2 release. In our experimental setting, the potassium ionophore nigericin was as potent as PMA in inducing RNASET2 release. Besides being a potent NET inducer, nigericin is an activator of NLRP3 inflammasome, a step required for IL-1β secretion in LPS-primed human blood neutrophils^24^. We found that nigericin alone was sufficient to trigger significant RNASET2 secretion and this effect was not potentiated by LPS priming or affected by the NLPR3 inhibitor MCC950, suggesting that RNASET2 release is independent of classical inflammasome stimulation^45^.

Other NET inducers, such as A23187, LPS, TNFα or fMLP were not able to induce RNASET2 release. Furthermore, recent proteomic analysis indicates that NETs triggered by different stimuli are heterogeneous in terms of protein composition^46^. Our results can be explained by the different ROS and calcium influx requirements of the various NET-inducer signalling pathways and suggest that RNASET2 is released and associates with NETs in response to certain NET inducing stimuli preferentially linked to “suicidal” NETosis. Moreover, the inability of fMLP, a powerful activator of PMN degranulation, to induce RNASET2 secretion, also leads to the assumption that RNASET2 is released via NETosis rather than degranulation. A similar pattern of secretion has been shown for other molecules, such as S100A8/A9^47^.

ICs are potent inducers of neutrophils activation through the engagement FcγRs, which play an important role in IC-mediated inflammatory process and development of autoimmune disease^48,49^. ICs not only occur as circulating sIC but they can be immobilized on extracellular surface. Both types of ICs can induce NETosis and, here we show that both types can stimulate the release of RNASET2 associated with extracellular traps. Of note, in support of this finding, we observed RNASET2 release following FcγRIIIb cross-linking that induces NET formation similar to PMA stimulation^50^.

Recent genome-wide association studies (GWAS) identified RNASET2 among novel genes associated with RA^15^. Among immune cells implicated in the pathophysiology of RA, neutrophils are the most abundant leukocytes in inflamed joints and possess the greatest cytotoxic potential and immunoregulatory role^16^. Taken together, these findings prompted us to assess the association of RNASET2 with neutrophils in RA. We demonstrate that RNASET2 is expressed in PMN and macrophages infiltrating the murine joints at the peak of K/BxN serum-transfer-induced arthritis, which models the effector phase of human RA. Co-localization of RNASET2 immune reactivity with CD163-positive macrophages and CD66b^+^ neutrophils was also present in synovial biopsies of RA patients with active disease. Given the increased expression within rheumatoid synovial tissue, we expanded the study to look at RNASET2 serum concentrations in STIA and CIA experimental murine RA models, which are feasible for studying immune system alterations during arthritis development. We found that the serum levels of RNASET2 were elevated after the induction of the two arthritis models, correlating with the onset and establishment of the disease. The highest circulating levels of RNASET2 were observed at the peak phase of disease severity and declines in the late stages in CIA and more rapidly in the STIA model paralleling the clinical features of the experimental arthritis. These findings were further supported by our pilot study in RA patients which showed significant increase in RNASET2 circulating levels compared with healthy subjects. In line with an association of RNASET2 with various autoimmune-mediated diseases, a recent work has reported increased RNASET2 levels in newly diagnosed Graves’ disease patients compared to healthy donors^51^.

The circulating levels of RNASET2 resulted increased in our cohort of RA patients compared with healthy controls, mirroring the behaviour of a prototypical inflammatory cytokine, IL-6, which plays a central role in the pathogenesis of RA^52^. In addition, a positive correlation, albeit weak, between IL-6 and RNASET2 was observed in our clinical setting.

Despite the high levels of RNASET2 in RA patients, no direct correlation was detected between RNASET2 and CRP nor DAS28-CRP disease activity score. This observation deserves further investigation due to some limitations of our study. Our cohort includes only patients under treatment with multiple cs- and b/tsDMARDs and no comparison was made with early-onset RA prior to treatment initiation. It is conceivable that RNASET2 levels may have been partially affected by the ongoing therapy. Larger validation studies in different subsets of RA patients, as well as the comparison with other forms of chronic arthritis and connective tissue disorders, are needed. In RA, the efforts should be aimed at investigating whether circulating RNASET2 can be included among surrogate biomarkers that could be used to predict RA synovial tissue phenotypes and the corresponding therapeutic response^53^. The colocalization of RNASET2 with neutrophils and macrophages in the synovial tissue might indicate that RNASET2 is involved in the active phase of synovitis since the early stages of disease. These results are relevant considering that synovial tissue research is an expanding field^54^ aimed at elucidating the role of different cell types in driving disease pathogenesis, at better stratifying patients, discovering biomarkers and designing novel target therapeutic approaches^55^. The local expression of RNASET2 may play a functional role in the induction and progression of joint inflammation and further studies are needed to evaluate its potential role as a prognostic marker of disease severity and/or response to treatment.

Our study provides a new insight into the contribution of PMN-associated RNASET2 into the pathogenesis of RA. Several reports point to an alarmin-like role for human RNASET2, which might act as a stress-induced secreted signalling molecule involved in sending a “danger” message to the innate immune system. As shown by Baranzini et al. in a leech model of infection, RNASET2 might act mainly as a migration signal to attract new macrophages, which in turn produce RNASET2 by themselves, to further strengthen the inflammatory state^56^. Given the close proximity of PMN and macrophages found in inflamed joint we can speculate that the RNASET2 release potentiated by PMN NETosis contributes to the recruitment of macrophages and that RNASET2 is involved in the active phase of synovitis since the early stages of disease. The increased serum levels found in RA patients and in mouse models of RA may be partially due to dysregulated NETosis, which triggers and amplifies inflammatory response. However, it should be considered that necrotic cells may also contribute to the total circulating levels of RNASET2. Taking together, neutrophil-derived RNASET2 may represent a new player in the complex pathogenetic network leading to autoimmune pathologies and a marker of systemic inflammation.

## Materials and methods

### Neutrophil Isolation and cultures

Neutrophils were isolated from peripheral blood of HC using Ficoll-Paque ^TM^ PLUS (Cytiva) gradient centrifugation. The granulocytic fraction was subjected to ACK Lysing Buffer (ThermoFisher Scientific), to lysis red blood cells, yielding a neutrophil population with a purity higher than 90%, as assessed by flow cytometry using CD16/CD66b staining (Miltenyi Biotec). For some experiments, higher neutrophil purity (99%) was obtained using the MACSxpress^®^ Whole Blood Neutrophil Isolation Kit (Miltenyi Biotec.). Immediately after purification, PMN were resuspended in RPMI 1640 medium supplemented with 10% heat-inactivated endotoxin-free FBS, 2 mM L-glutamine, penicillin, and streptomycin (all from Gibco, Thermo Fisher Scientific) and plated in 24 or 48 well plates maintaining a concentration of 5 × 10^6^/ml or plated at 1 × 10^6^/ml in 96 well plates for experiments regarding neutrophils activation by immune complexes (IC) and FcγRIIIb (CD16) crosslinking. PMN were warmed to 37 °C and then stimulated with Phorbol 12-Myristate 13-Acetate (PMA, 100 ng/ml, Sigma), Nigericin (15µM; Adipogen Life Sciences), A23187 (5µM; Calbiochem), LPS (100 ng/ml; Sigma), TNF-α (20 ng/ml; Miltenyi Biotec), fMLP (100 nM; Sigma) for the indicated times. In selected experiments, PMN were pretreated with DPI (10 µM; Adipogen Life Sciences) for 30 min before PMA stimulation for 4 h. In other sets of experiments PMN were LPS primed (500 ng/ml) for 2 h followed by Nigericin treatment (15 µM) for 2 h in presence/absence of MCC950 (10µM, Adipogen Life Sciences), added 15 min before Nigericin stimulation. Neutrophils activation by IC and CD16 crosslinking was performed as described below. After incubation, neutrophils were centrifuged, and cell-free supernatants were collected for ELISA assays.

### Immunofluorescent analysis of neutrophils granules

Freshly isolated PMN were cytospinned for 5 min at 900 rpm and then fixed with 4% paraformaldehyde (Pierce, Thermo Fisher Scientific). Specimens were permeabilized and blocked with 0.1% Triton X-100/PBS with 50 µg/mL of human IgG and stained with primary antibodies: anti-MPO (CloudClone Corp.) at dilution of 1:50, anti-MMP9 (Dianova GmbH) at dilution of 1:20, anti-lactoferrin (Nordic-MUbio) at dilution of 1:200, anti-RNASET2 at dilution of 1:100 (LifeSpan BioSciences, Inc.). Alexa fluor 594 goat anti-rabbit (ThermoFisher Scientific) used as secondary antibody for RNASET2. Alexa fluor 488 goat anti-mouse (ThermoFisher Scientific) was used for MPO, MMP-9 and lactoferrin. DAPI (ThermoFisher Scientific) was used to stain the nuclei.

### Computational analysis of RNA-seq datasets

RNA-seq datasets from neutrophil progenitors^26^ were downloaded from Gene Expression Omnibus database (http://www.ncbi.nlm.nih.gov/geo/) under accession number GSE164687. Computational analysis of transcriptome datasets generated by Smart-seq2^27^ has been performed using the bioinformatic pipeline utilized in a previous study^28^, with minor modifications. Briefly, binary base call (BCL) files generated by the Illumina sequencer were converted into FastQ files using bcl2fastq v2.20 software. After quality filtering, according to the Illumina pipeline, the contaminant adapters in the FastQ files were detected using FastQC v0.11.9. Then, adapters removal and base quality trimming were performed using Trim Galore! (http://www.bioinformatics.babraham.ac.uk/projects/trim_galore/) script with the length parameter set to 50. Trimmed reads were quantified using Kallisto quant applying parameters -bias -single -l 200 -s 20 and human transcriptome reference ensemble version 96 (http://apr2019.archive.ensembl.org/index.html). Transcript quantification obtained from Kallisto was combined to gene level using tximport packages v1.22.0. Gene counts were normalized among various samples using DESeq2 v1.34.0, and only genes coding to protein and long non-coding RNA (lnRNA) were retained for downstream analysis. Transcript abundance was indicated as Fragments Per Kilobase of transcript sequence per Million of mapped fragments (FPKM), using the “fpkm” function of DESeq2.

### NET release

 PMN (5 × 10^6^ cell/mL) were seeded on polylysine-coated glass slides and left adhering for 30 min at 37 °C. Then, PMN were treated for 1 h with PMA (100 ng/mL) or soluble IC (sIC). Next, cells were fixed with 1% paraformaldehyde, and incubated with a rabbit anti-citrullinated–histone H4 antibody (Abcam) or with rabbit polyclonal antibody anti-RNASET2 (LifeSpan BioSciences, Inc.), followed by an Alexa-488- conjugated anti-rabbit antibody (Thermo Fisher Scientific). Hoechst 33,342 dye was used to stain cell DNA. NETs were observed with the fluorescence microscopy Zeiss Observer.Z1 at a magnification of 200x and Apotome2 for optical sectioning. Images were acquired using AxioVision software. The quantification of colocalization of RNASET2 with neutrophil granule proteins was expressed as colocalization coefficient, which indicates the relative number of colocalized pixels in the RNASET2 channel in relation to the total number of pixels in the channel of the granule protein, above the threshold value.

### Neutrophils activation by IC and CD16 cross-linking

IC were prepared by mixing human serum albumin (HSA, Sigma) and rabbit polyclonal anti-HSA IgG (anti-HSA, Sigma) as previously described^18,19^. The point of equivalence, that is the optimal antigen: antibody ratio required for the formation of insoluble complexes, was determined by measuring the absorbance at 450 nm. sIC were prepared at sixfold the concentration of antigen required to form insoluble complexes. After incubation at 37 °C for 1 h with gently agitation, sIC were briefly centrifuged (5 min at 3000 rpm), supernatant was decanted and centrifuged again before use. 10 µl of sIC were added to 100 µl neutrophil suspension. PMN were stimulated in parallel with relative controls (HSA and anti-HSA) for 4 h.

For the preparation of plate-bound immobilized IC (iIC), 96 well high-binding plates were coated overnight at 4 °C with 20 µg/ml HSA in 50 mM carbonate/bicarbonate buffer pH 9.6. Plates were washed with PBS 0.05% Tween and then blocked with 10% BSA in PBS for 1 h at room temperature, washed and incubated for 1 h with anti-HSA IgG (1:800 in PBS, about 18 µg/ml IgG). After washing PMN activation by iIC was achieved by plating PMN on the IC-coated surfaces for 4 h.

PMN were stimulated by CD16 cross-linking with specific mAbs as follows: PMN were incubated at 4 °C for 30 min with anti-human CD16b (clone 3G8; Novus Biologicals) at the concentration of 1 µg/10^6^ cells and washed with PBS. For the cross-linking, cells were then treated with 1 µg/10^6^ cells F(ab′)_2_ of goat anti-mouse Ig (GAM) (Jackson ImmunoResearch Europe Ltd) at 37 °C for 4 h.

### Animals

Age- and sex-matched mice were used for experiments. Procedures involving animals handling were conformed to institutional guidelines in compliance with national (D.L. N.26, 4-3-2014) and international (Directive 2010/63/EU revising Directive 86/609/EEC, September 22, 2010) law and policies. The study was approved by the Italian Ministry of Health (approval number 144/2020-PR). All efforts were made to minimize the number of animals used and their suffering. Animal use followed the recommendation of the ARRIVE guidelines.

### Induction of experimental arthritis and serum collection

Our study examined male and female animals, and similar findings are reported for both sexes. Collagen-induced arthritis (CIA) was induced in 8- to 12-week-old male DBA1 mice with 100 mg of denatured type II bovine collagen (MD Biosciences) emulsified in complete Freund’s adjuvant^35^. Serum was collected before collagen injection and after 1, 31, 74 days. For the induction of serum transfer–induced arthritis (STIA), C57BL/6J mice were intraperitoneally injected with 150 mL of serum from K/BxN transgenic mice^34^. Serum was collected before K/BxN serum injection and after 6, 10, 24 days.

### Clinical assessment of experimental arthritis

All mice were examined two to three times per week for the initial visual appearance of arthritis after immunization. Arthritis of each individual limb was graded using the following scoring system: 0, normal; 1, apparent swelling and redness limited to individual digits; 2, swelling in more than one joint; 3, severe redness and swelling of the entire paw including digits; and 4, maximally inflamed limb with involvement of multiple joints. The maximum score per mouse was 16. Mice were scored as arthritic if more than one paw had a score > 2. The thickness of the hind paws was measured using a dial gauge caliper (Mitutoyo).

### Patients

One hundred-nine consecutive patients with RA, followed-up at the Rheumatology and Clinical Immunology Unit, ASST Spedali Civili in Brescia, were enrolled in the study. Their main demographic and clinical features are shown in Table 1. The study was approved by the Institution Ethics Committee of ASST Spedali Civili in Brescia (approval number NP3780). According to the Declaration of Helsinki, written informed consent was obtained from all subjects with regard to blood and tissue samples. Clinical disease activity was evaluated with the Disease Activity Index 28 based on C-Reactive Protein (CRP) (DAS28-CRP)^57^ and patients were divided into three groups for analysis based on disease activity (moderate/high, low and remission).

**Table 1:**
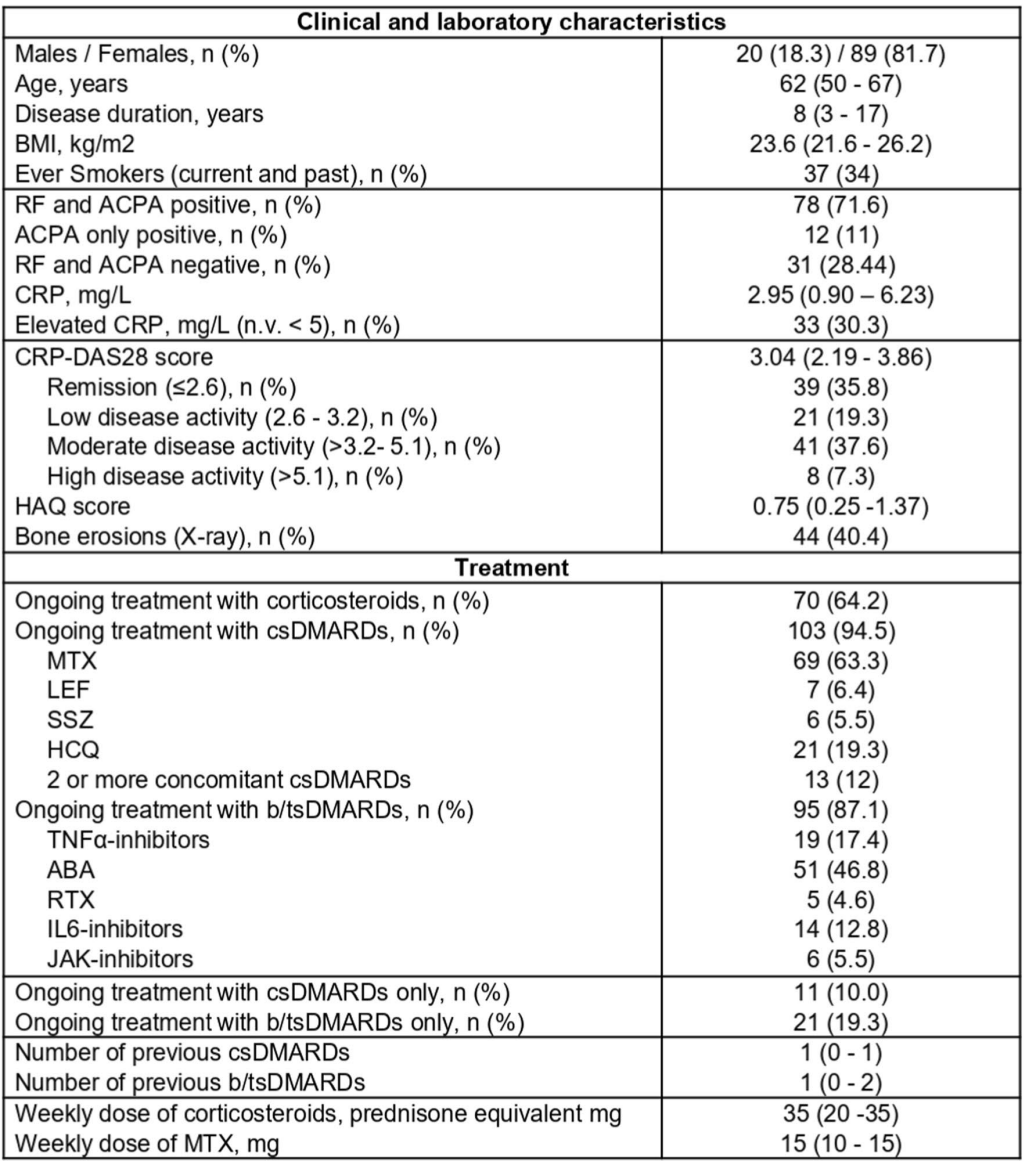
Clinical, laboratory and treatment features of enrolled patients with Rheumatoid Arthritis. Results are shown as median (IQR), if not otherwise specified. Abbreviations: ABA: abatacept; ACPA: anti-citrullinated peptides antibodies; BMI: body mass index; b/ts: biological/target synthetic; disease modifying anti-rheumatic drugs; HAQ: health assessment questionnaire; HCQ: hydroxychloroquine; IL: interleukin; LEF: leflunomide; MTX: methotrexate; n.v.: normal value; RF: rheumatoid factor; RTX: rituximab; SSZ: sulfasalazine.

Fifty-three sex- and age-matched healthy controls (HC) (females, 71.7%) with a median age (25th-75th percentile) of 53 (48–58) were also enrolled.

Serum samples were collected from RA patients and HC by means of standard peripheral venepuncture and then stored at -80 °C.

### ELISA assays

Human RNASET2 and IL-6 concentrations in sera from HC and RA patients were determined using ELISA commercial kits (Fine Test and R&D respectively) with sensitivity limits of 0.188 ng/mL for RNASET2 assay and 9.4 pg/mL for IL-6 assay. RNASET2 kit was used for cell-free supernatants from in vitro stimulated PMN. Circulating levels of murine RNASET2 were determined by a commercially available ELISA assay (LSBio Life Span Biosciences nc.) with sensitivity limits of 0.125 ng/ml. ELISA kit from R&D Systems was used to quantify IL-1β levels in human PMN culture supernatants.

### Immunohistochemistry

RNASET2 expression was tested on human and mouse tissues by using anti-RNASET2 polyclonal rabbit antibodies (HPA029013, 1:100, Sigma and LS-B8807, 1:1000, LSBio Life Span Biosciences Inc., respectively). Human synovial biopsies were collected from the archive of the Pathology Unit, ASST Spedali Civili in Brescia. Mouse joints deriving from STIA-induced mice were removed, fixed, and decalcified. Paraffin-embedded tissue blocks were cut with a microtome into fine slivers of 3 microns. Briefly, anti-RNASET2 were revealed using Novolink Polymer (Leica) or EnVision + System-HRP Labelled Polymer Anti-Rabbit (Dako) followed by DAB. For double staining, anti-RNASET2 was combined with anti-CD66b (clone G10F5, 1:150, BioLegend), anti-CD163 (clone 10D6, 1:50, Neomarkers), anti-IBA1 (polyclonal Rabbit, 1:300, Wako) and anti-LY6G (clone 1A8, 1:400, Cederlane). After completing the first immune reaction, the second immune reaction was visualized using Mach 4 MR-AP (Biocare Medical) or Rat-on-Mouse AP-Polymer (Biocare Medical), followed by Ferangi Blue.

### Statistical analyses

Categorical and continuous variables were expressed as percentage and as median value, respectively. Differences between groups were evaluated by Mann-Whitney U-test, as appropriate. Statistical analyses of RNASET2 concentrations in human and murine serum were performed using Student *t* test and one-way analysis of variance (ANOVA), as appropriate. Statistical analysis of stimulated PMN were performed using ANOVA p value ≤ 0.05 was considered statistically significant. The Spearman test was adopted for correlation analysis, and *p* < 0.05 was considered significant. Results were analysed by using GraphPad PRISM 8.0 software.

## Electronic supplementary material

Below is the link to the electronic supplementary material.

Supplementary Material 1

## Data Availability

The bulk RNAseq datasets analysed during the current study are available in the Gene Expression Omnibus database (http://www.ncbi.nlm.nih.gov/geo/) under the accession number GSE164687. The other data supporting this study’s findings are available from the corresponding author upon reasonable request.

## Abbreviations

PMN: Neutrophils
RA: Rheumatoid Arthritis
STIA: Serum Transfer Induced Arthritis
CIA: Collagen Induced Arthritis
NET: Neutrophil Extracellular Trap
GWAS: Gene Wide Association Studies
FcRs: Fc receptors
ROS: Reactive Oxygen Species
RNS: Reactive Nitrogen Species
IC: Immune complexes
HC: Healthy Controls
HSA: Human Serum Albumin
PMA: Phorbol 12-Myristate 13-Acetate
LPS: Lipopolysaccharide
TNF: Tumor Necrosis Factor
fMLP: N-Formylmethionyl-leucyl-phenylalanine
GAM: Goat Anti-Mouse Ig
MPO: Myeloperoxydase
MMP: Matrix metalloproteinases
DAPI: 4′,6-diamidino-2-phenylindole
DPI: diphenyliodonium
FPKM: Fragments Per Kilobase of transcript sequence per Million of mapped fragments
IHC: Immunohistochemistry
IF: Immunofluorescence
qRT–PCR: Quantitative real-time PCR
DAS28: Disease Activity Score 28
CRP: C-Reactive Protein
IL-1β: Interleukin 1β
IL-6: Interleukin 6
