# Author Correction: Regulation of neutrophil associated RNASET2 expression in rheumatoid arthritis

**DOI:** 10.1038/s41598-024-79660-0

**Published:** 2024-11-19

**Authors:** Mauro Passari, Sara Scutera, Tiziana Schioppa, Laura Tiberio, Silvia Piantoni, Nicola Tamassia, Mattia Bugatti, William Vermi, Fabrizio Angeli, Alessia Caproli, Valentina Salvi, Francesca Sozio, Angela Gismondi, Helena Stabile, Franco Franceschini, Daniela Bosisio, Francesco Acquati, Sonja Vermeren, Silvano Sozzani, Laura Andreoli, Annalisa Del Prete, Tiziana Musso

**Affiliations:** 1 Department of Molecular and Translational Medicine, University of Brescia, Viale Europa 11, Brescia, 25123 Italy; 2 Department of Public Health and Pediatrics, University of Turin, Turin, Italy; 3 IRCCS Humanitas Research Hospital-Rozzano, Milan, Italy; 4 Department of Clinical and Experimental Sciences, Unit of Rheumatology and Clinical Immunology - ASST, University of Brescia, Spedali Civili of Brescia, Brescia, Italy; 5 Department of Medicine, Section of General Pathology, University of Verona, Verona, Italy; 6 Department of Molecular Medicine, Laboratory Affiliated to Istituto Pasteur Italia- Fondazione Cenci Bolognetti, Sapienza University of Rome, Rome, Italy; 7 Human Genetics Laboratory, Department of Biotechnology and Life Sciences, University of Insubria, Varese, Italy; 8 Centre for Inflammation Research, Institute for Regeneration and Repair, The University of Edinburgh, Edinburgh, UK

Correction to: *Scientific Reports* 10.1038/s41598-024-77694-y, published online 05 November 2024

In the original version of this Article, the author name Annalisa Del Prete was incorrectly indexed. The original Article has been corrected.

