# Supplementary material for: Regulation of neutrophil associated RNASET2 expression in rheumatoid arthritis
Source: Sci Rep. 2024 Nov 5;14:26820. doi: 10.1038/s41598-024-77694-y (PMC11538310; doi:10.1038/s41598-024-77694-y)
Supplement: Supplementary file 1 — Supplementary Material 1 [file 41598_2024_77694_MOESM1_ESM.pdf]

A

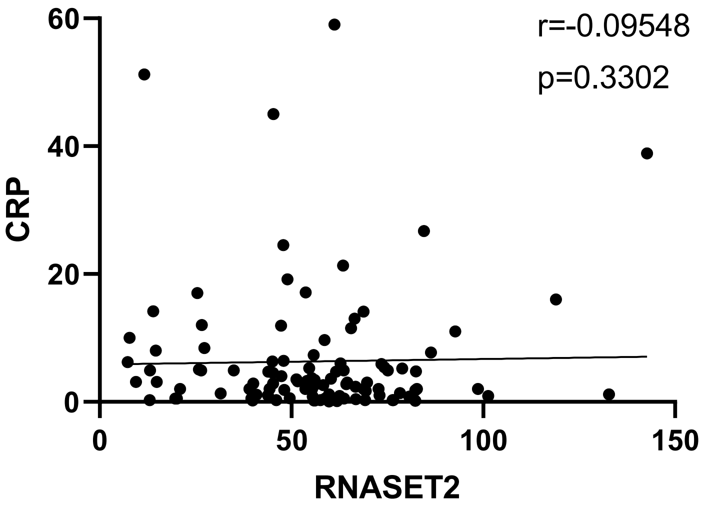

B

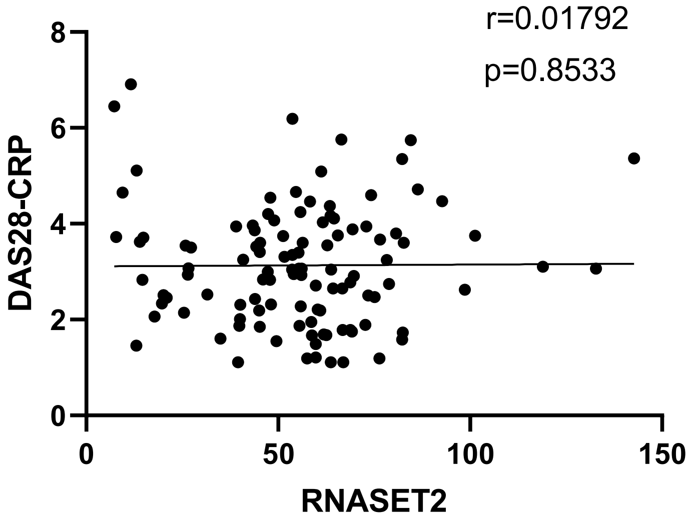

Supplementary Figure 1 - Correlation of RNASET2 circulating levels with disease activity indexes. Correlation between RNASET2 serum levels and CRP **(A)** and DAS28-CRP **(B)** by Spearman test.
